# Bilberry Anthocyanins Ameliorate NAFLD by Improving Dyslipidemia and Gut Microbiome Dysbiosis

**DOI:** 10.3390/nu12113252

**Published:** 2020-10-23

**Authors:** Hironobu Nakano, Shusong Wu, Kozue Sakao, Taichi Hara, Jianhua He, Santos Garcia, Kalidas Shetty, De-Xing Hou

**Affiliations:** 1 Graduate School of Agriculture, Kagoshima University, Kagoshima 890-0065, Japan; k8108910@kadai.jp (H.N.); sakaok24@chem.agri.kagoshima-u.ac.jp (K.S.); 2 College of Animal Science and Technology, Hunan Agricultural University, Changsha 410128, China; wush688@126.com (S.W.); jianhuahy@hunau.net (J.H.); 3 The United Graduate School of Agricultural Sciences, Kagoshima University, Kagoshima 890-0065, Japan; 4 Faculty of Human Sciences, Waseda University, 2-579-15 Mikajima, Tokorozawa, Saitama 359-1192, Japan; harata1@waseda.jp; 5 Fac. C. Biologicas, Universidad Autonoma de Nuevo Leon, San Nicolas 66451, Mexico; santos@microbiosymas.com; 6 Department of Plant Science, North Dakota State University, Fargo, ND 58105, USA; Kalidas.Shetty@ndsu.edu

**Keywords:** bilberry, anthocyanins, NAFLD, dyslipidemia, gut microbiome

## Abstract

Non-alcoholic fatty liver disease (NAFLD) is a manifestation of metabolic syndrome closely linked to dyslipidemia and gut microbiome dysbiosis. Bilberry anthocyanins (BA) have been reported to have preventive effects against metabolic syndrome. This study aimed to investigate the protective effects and mechanisms of BA in a Western diet (WD)-induced mouse model. The results revealed that supplementation with BA attenuated the serum levels of aspartate aminotransferase (AST), alanine aminotransferase (ALT), low-density lipoprotein cholesterol (LDL-c), fat content in liver, 2-thiobarbituric acid reactive substances (TBARS) and α-smooth muscle actin (α-SMA) caused by WD. Furthermore, gut microbiota characterized by 16S rRNA sequencing revealed that BA reduced remarkably the ratio of Firmicutes/Bacteroidetes (F/B) and modified gut microbiome. In particular, BA increased the relative abundance of g_*Akkermansia* and g_*Parabacteroides*. Taken together, our data demonstrated that BA might ameliorate WD-induced NAFLD by attenuating dyslipidemia and gut microbiome dysbiosis.

## 1. Introduction

Non-alcoholic fatty liver disease (NAFLD) is a condition in which excess fat is stored in the liver without significant alcohol consumption. It embraces a pathological spectrum from simple steatosis to steatohepatitis; 10–20% have steatohepatitis (non-alcoholic steatohepatitis: NASH), which can progress to cirrhosis and hepatocellular carcinoma [1,2]. NAFLD prevalence is reported from 21% to 24.7% in USA and North America, 24% in Europe, and from 23% to 26% in Japan [3]. NAFLD is closely associated with type 2 diabetes and obesity, and both of these conditions drive progressive disease toward the more advanced stages. The mechanisms that govern hepatic lipid accumulation and the predisposition to inflammation and fibrosis are still not fully understood but reflect a complex interplay between metabolic target tissues including adipose, immune and inflammatory cells [4]. These events cause the accumulation of fat in the liver but whether the liver will become a fatty liver depends on the magnitude of these events. The sequence of the events is fatty liver, then NAFLD, and then steatohepatitis (NASH) [4,5]. In particular, a high-fat diet results in overgrowth of some intestinal bacteria, choline-deficiency, ethanol production and disruption of intestine tight junction, finally leading to the invasion of microbial products such as lipopolysaccharide, trimethylamine, and ethanol to the liver [6]. Thus, gut microbiome dysbiosis caused by a high-fat diet is considered as an important aspect of NAFLD progression [6].

There is no established treatment for NAFLD although several lines of study have reported that insulin sensitizers such as thiazolidinediones and antioxidants such as vitamin E can improve clinical and histologic features of NAFLD [7]. Furthermore, a number of phase 3 clinical trials are currently ongoing including Elafibranor (a dual peroxisome proliferator-activated receptor (PPAR) α/δ agonist), Cenicriviroc (a C-C chemokine receptor 2 (CCR2)/C-C chemokine receptor 5 (CCR5) antagonist), Obeticholic acid (the nuclear bile acid receptor Farnesoid X receptor (FXR) agonist), Aramchol (a fatty acid bile acid conjugate that modulates stearoyl-CoA desaturase(SCD)-1), and Resmetrion (a liver-specific thyroid hormone receptor (THR)-β agonist) [8]. These pharmacotherapies are expected to become available in the near future, none to date has been approved [8]. Thus, NAFLD prevention by dietary administration has recently been considered an important strategy.

*Vaccinium myrtillus* L. also known as bilberry, is a small edible berry that grows on small slender shrubs on hillsides throughout central and northern Europe [9]. Bilberry has 15 species of anthocyanins with 5 species of anthocyanidin (delphinidin, cyanidin, petunidin, peonidin and malvidin) and 3 kinds of glycoside (galactoside, glucoside and arabinoside) [10]. Bilberry anthocyanins powder, defined as bilberry anthocyanins (BA) in this study or as Mirtoselect in other studies, contains high concentration of anthocyanins (>36%). It has been reported that BA or Mirtoselect has antioxidant effects [11], and could attenuate type 2 diabetes [12]. Moreover, Mirtoselect has been recently reported to attenuate NASH and associated fibrosis in ApoE*3Leiden (E3L) mice [13]. The mechanisms are suggested that Mirtoselect attenuated development of NASH through the reduction of hepatic lipid accumulation, inflammation and fibrosis, possibly mediated by local anti-inflammatory effects associated with reduced accumulation and crystallization of intrahepatic free cholesterol [13]. However, the effects of Mirtoselect on gut microbiome were not investigated, and the relationship between NASH-related symptoms and gut microbiome changes by Mirtoselect is unclear. Moreover, in a previous study, a translational female E3L mice model was used to investigate the treatment effects of Mirtoselect on NASH because all mice received Western diets (WD) for 4 weeks, and only the positive mice who matched the serum levels of cholesterol and triglycerides were selected to further feed with Mirtoselect [13].

Based on this information, in the present study we used normal male mice (male C57BL/6N mice) and induced NAFLD by 3-months intake with WD to mimic natural NAFLD development. At the same time, we supplied BA in diet from the beginning of WD intake. Thus, our animal model in the present study is to estimate the preventive effects of BA by dietary supplemental administration, Thus, we first investigated serum and hepatic markers regarding lipid accumulation, inflammation and oxidative status to confirm our mice model effect for NAFLD induction, and then focused on investigating microbiome changes by BA supplementation, and further discussed the relationship between microbiome changes and NAFLD attenuation by BA.

## 2. Materials and Methods

### 2.1. Chemicals and Reagents

Bilberry anthocyanins extract powder (Mirtoselect) was purchased from Indena Japan Co., Ltd. (Tokyo, Japan) and batch number was 32265/M2. Lard and cellulose were purchased from Sigma-Aldrich Co., LLC. (Tokyo, Japan). Soybean oil, cholesterol, choline bitartrate, methionine, fructose, acetic acid, lactic acid, propionic acid, 1,1,3,3-tetraethoxypropane, 2-thiobarbituric acid, and proteinase inhibitor cocktail were purchased from Nacalai Tesque, Inc. (Kyoto, Japan). AIN-93G mineral mix and AIN-93G vitamin mix were purchased from Oriental Yeast Co., Ltd. (Tokyo, Japan). Corn starch was purchased from Sanwa Starch Co., Ltd. (Nara, Japan). Edible Acid Casein 30–60 Mesh was purchased from Meggle GmbH & Co. KG (Wasserburg am Inn., Germany). Sucrose was purchased from Hayashi Pure Chemical Industry., Ltd. (Osaka, Japan). Paraformaldehyde, hexane and 2-ethyl butyric acid were purchased from Wako Pure Chemical Co. (Osaka, Japan). Oil red O reagent was purchased from Muto Pure Chemicals Co., Ltd. (Tokyo, Japan). Antibodies against NF-E2-related factor 2 (Nrf2), Kelch-like ECH-associated protein 1 (Keap1), superoxide dismutase-2 (SOD2), and α-smooth muscle actin (α-SMA) were purchased from Santa Cruz Biotechnology (Santa Cruz, CA, USA). Antibodies against β-actin were purchased from Cell Signaling Technology (Beverly, MA, USA).

### 2.2. Animal Experiment Design

The animal experimental protocol was drafted according to the Guidelines of the Animal Care and Use Committee of Kagoshima University (Permission NO. A12005). Male C57BL/6N mice (5 weeks age) from Japan SLC Inc. (Shizuoka, Japan) were housed separately in cages with wood shavings bedding under controlled light (12 h light/day) and temperature (23.5 °C), and free access to water and feed. After acclimatization for 1 week, the mice were randomly divided into 4 groups (*n* = 5): normal diet (ND) group, ND + 2% BA group (NDBA), Western diet (WD) group, WD + 2% BA group (WDBA) (Appendix A Table A1). ND contained 3% lard and 3% soybean oil, WD contained 30% lard, 3% soybean oil and 1.5% cholesterol. Normal water was provided to ND and NDBA groups, and 4% fructose water was provided to WD and WDBA groups [14]. The supplemented dose of BA was determined according to our preliminary study without toxicity (data not shown). Mice were sacrificed at 18 weeks age after overnight fasting.

### 2.3. Measurement of Serum Biochemical Indexes

Blood was obtained from mice orbital veins and collected into a tube with coagulant (Separable microtubes, FUCHIGAMI, Kyoto, Japan) for 30 min at room temperature. Serum were separated from blood after centrifugation at 4000 rpm for 5 min and stored at −80 °C until use. The serum levels of alanine transaminase (ALT), aspartate aminotransferase (AST), total cholesterol (T-Cho), total triacylglycerol (TG), high density lipoprotein cholesterol (HDL-c), and glucose were measured with an automated analyzer for clinical chemistry (SPOTCHEM EZ, Arkray, Kyoto, Japan). The level of low-density lipoproteins (LDLs) was calculated using the Friedewald equation (LDL = T-Cho - HDL-c - TG/5) [15]. The serum concentrations of insulin and monocyte chemotactic protein-1 (MCP-1) were measured with an enzyme-linked immunosorbent assay (ELISA) kit (Invitrogen, San Diego, CA, USA) according to the manufacturer’s instructions. The index of homeostatic model assessment for insulin resistance (HOMA-IR) was calculated with the function of fasting glucose × fasting insulin/405 [16].

### 2.4. Measurement of 2-Thiobarbituric Acid Reactive Substances (TBARS)

The liver TBARS concentration was measured according to the previous method modified slightly [17]. The reaction mixture contained 30 μL of liver homogenate (liver:PBS = 1:10), 30 μL of 8.1% sodium dodecyl sulfate (SDS), 225 μL of 20% acetic acid, 225 μL of 0.8% thiobarbituric acid. After incubation for 60 min at 100 °C, 150 μL of water was added and soon moved on to ice to stop the reaction. After cooling, 750 μL of the butanol-pyridine mixture (15:1) was added to the reaction mixture, and then centrifuged at 8000 rpm for 10 min. The absorbance of supernatant was read at 532 nm. The concentration of the TBARS was expressed in nmol/mg liver proteins. The range of 10~100 μM of 1,1,3,3-tetraethoxypropane was used as the standards.

### 2.5. Histomorphological Analysis

Mouse liver was collected at 18 weeks and fixed in 4% paraformaldehyde dissolved in phosphate buffered (PBS) at 4 °C overnight. After fixation, the liver was washed with PBS and preserved at 4 °C in 20% sucrose dissolved with PBS overnight. After dehydration, liver was mounted with O.C.T. Compound (Sakura Finetek Japan Co., Ltd., Tokyo, Japan) under dry ice -isopentane, and preserved at −80 °C. The mounted liver was sliced at 5 μm by microtome (Yamato, Saitama, Japan). The sliced liver section was stained by oil red O for 10 min, washed by 60% isopropanol, and then observed under a fluorescence microscope (Keyence, Tokyo, Japan).

### 2.6. Extraction of Hepatic and Fecal Lipid

One hundred mg of liver or feces from each mouse were homogenized in hexane (200 mg/mL) with a Speed-Mill PLUS homogenizer (Analytik Jena, Jena, Germany). The supernatant was obtained by centrifugation at 8000 rpm for 10 min at room temperature. Three hundred μL of supernatant were transferred to new tube and evaporated by centrifugal concentration equipment, the residue was then weighed.

### 2.7. Measurement of Short-Chain Fatty Acid (SCFA) Concentration in Caecum

One hundred mg of caecum contents from each were homogenized in MQ water (100 mg/mL) by vortex for 30 s, and then centrifuged at 8000 rpm for 10 min at room temperature to get supernatant. A mixture of 100 μL of the caecum supernatant and 200 μL of 0.25 mM 2-ethylbutyric acid as internal standard were labelled with 2-nitrophenyl hydrazide (2-NPH) using a short- and long-chain fatty acid analysis kit (YMC Co., Ltd., Kyoto, Japan) according to the manufacturer’s manual. 2-NPH labelled caecum supernatant was filtered through a 0.45 μm syringe filter (Millex Syringe Driven Filter Unit, MERCK, Darmstadt, Germany), and then injected into a high-performance liquid chromatography (HPLC) system (JASCO Corporation, Tokyo, Japan) with a YMC-Pack FA column (250 × 4.5 mm; YMC Co., Ltd. Tokyo, Japan). Mobile phase was acetonitrile-methanol-water (30:16:54, *v/v/v*), which adjusted to pH 4.5 by 0.01 M of hydrochloric acid. The injection volume was 20 μL and flow rate was kept at 1 mL/min and column oven temperature was set as 50 °C.

### 2.8. Hepatic Protein Extraction and Western Blot Assay

The total proteins of liver were extracted as described in our previous study [18]. Liver proteins (50 μg) were run on sodium dodecyl sulphate-polyacrylamide gel electrophoresis (SDS-PAGE) gel before transferring to poly vinylidene di-fluoride (PVDF) membrane (GE Healthcare, Buckinghamshire, UK). The membrane was incubated with specific primary antibody and then with horseradish peroxidase (HRP)-conjugated secondary antibody. The detection was performed with a LumiVision PRO system (TAITEC Co., Saitama, Japan).

### 2.9. Gut Microbiota Analysis by 16S rRNA Gene Sequencing

The feces DNA were extracted by FastDNA SPIN kit for Feces (MP Bio Japan K. K., Tokyo). The composition of gut bacterial communities was analyzed by sequencing 16S rRNA genes as described in our previous study [19]. The sequences were grouped in operational taxonomic units (OTUs) with 97% similarity by QIIME 2.0. Relative abundance is calculated as the percentage of a certain bacteria OTUs to the total number of OTUs.

### 2.10. Statistical Analysis

Significant differences between groups were determined by Duncan’s multiple range tests (IBM SPSS Statistics 24, IBM Japan, Ltd., Tokyo, Japan). A probability of *p* < 0.05 was considered significant. Pearson correlation was analyzed by IBM SPSS Statistics 24. The gplots package was used to draw a heatmap in RStudio (version 1.2.5001).

## 3. Results

### 3.1. Body Weight and Indexes of Liver Injury

**Figure A1:**
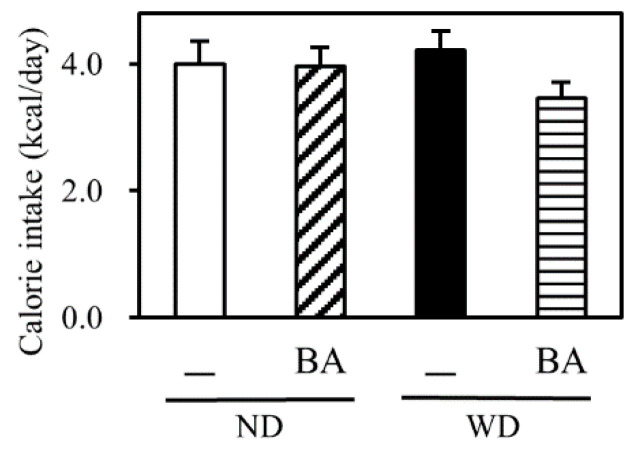
Effects of BA on calorie intake. Data are shown in mean ± SE of five mice for each group. ND: normal diet, WD: Western diet, BA: 2% bilberry anthocyanins.

The final body weight, liver weight, epididymis fat weight, the ratio of liver weight/body weight, liver fat weight as well as total feces fat of mice fed with WD at 18 weeks was significantly higher than the mice fed with ND (Table 1). Supplementation with BA decreased significantly WD-enhanced all of these items except epididymis fat weight (Table 1). The calories from food intake had no significant difference in all groups (Figure A1).

**Figure 1:**
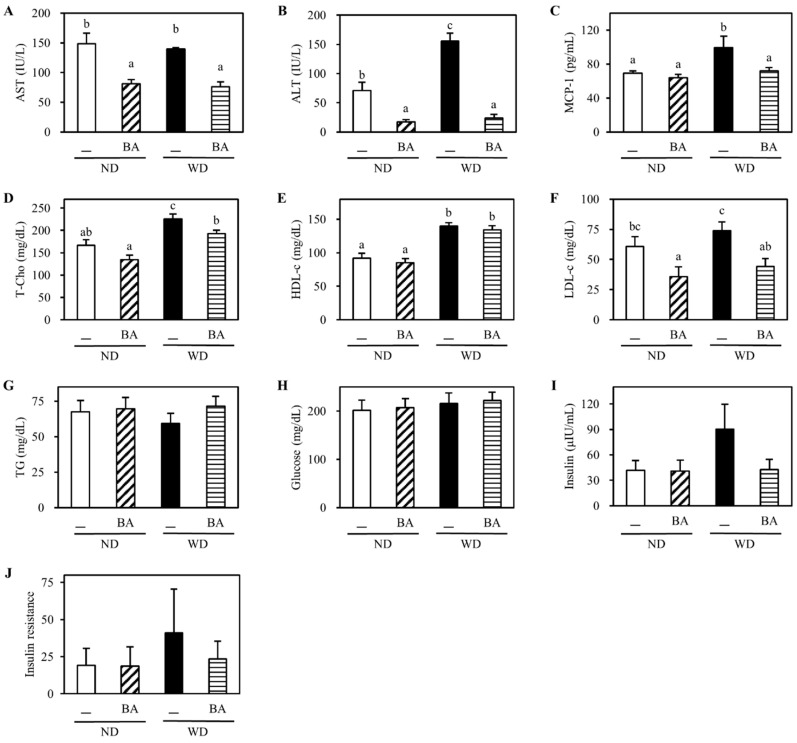
Effects of BA on serum levels of (**A**) aspartate aminotransferase (AST), (**B**) alanine aminotransferase (ALT), (**C**) monocyte chemotactic protein-1 (MCP-1), (**D**) total cholesterol (T-Cho), (**E**) high-density lipoprotein cholesterol (HDL-c), (**F**) low-density lipoprotein cholesterol (LDL-c), (G) total triacylglycerol (TG), (**H**) glucose, (**I**) insulin, and (**J**) insulin resistance. The data represent the mean ± standard error (SE) of five mice for each group. Columns with different letters significantly differ (*p* < 0.05). ND: normal diet, NDBA: ND + 2% BA, WD: Western diet, WDBA: WD + 2% BA.

The serum levels of liver injury indexes including AST (Figure 1A) and ALT (Figure 1B) were significantly decreased by supplementation with BA in both the ND and WD groups. The serum level of MCP-1 (Figure 1C), an inflammatory cytokine, was significantly increased in WD, and significantly reduced by supplementation with BA. The serum level of T-Cho (Figure 1D) was also significantly increased in WD, and significantly reduced by supplementation with BA. LDL-c (Figure 1F) was significantly decreased by supplementation with BA in both the ND and WD groups. However, the serum levels of HDL-c (Figure 1E), TG (Figure 1G) or glucose (Figure 1H) were not significantly different in all groups. Additionally, the serum levels of WD-enhanced insulin (Figure 1I) and insulin resistance (Figure 1J) showed reduced trend by supplementation with BA.

**Figure 2:**
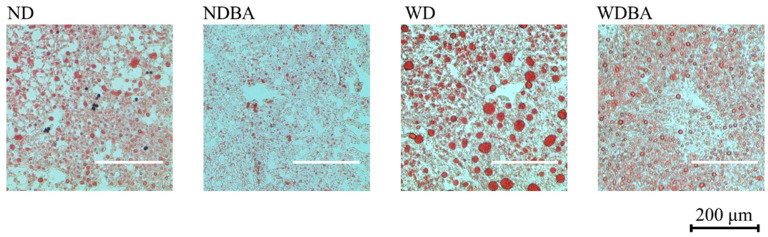
Influence of BA on liver fat morphology stained by Oil red O. Scale bar is 200 μm. ND: normal diet, NDBA: ND + 2% BA, WD: Western diet, WDBA: WD + 2% BA.

To assess fat accumulation and excretion, we investigated the liver lipid droplets stained by Oil red O, and simultaneously measured lipid contents in both liver and feces. Oil red O stain on the liver section showed that WD caused accumulation of many lipid droplets, which were reduced by supplementation with BA (Figure 2). Quantitative results revealed that fat content in the liver of WD was 3.6-fold higher than that of ND, and BA significantly decreased the fat contents, compared with ND and WD, respectively (Table 1).

### 3.2. Activation of Nrf2-Antioxidant Pathway and Amelioration of Non-Alcoholic Fatty Liver Disease (NAFLD)-Associated Markers

**Figure 3:**
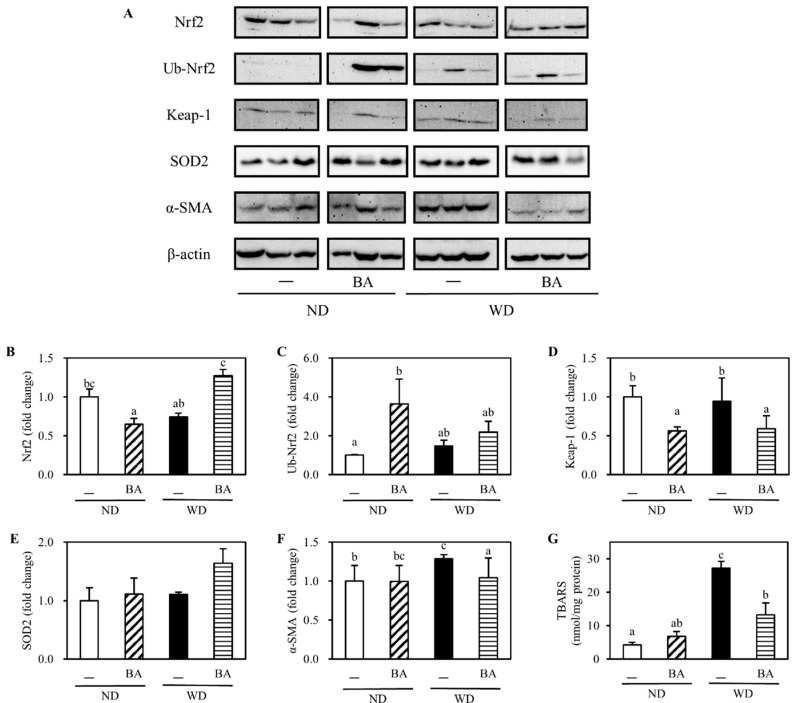
Effects of BA on induction of liver antioxidant and NAFLD-associated proteins. (**A**) Representative Western blots of the indicated proteins from three mice randomly selected from each group were shown in Figure 3**A**. The induction folds of (**B**) NF-E2-related factor 2 (Nrf2), (**C**) ubiquitinated Nrf2 (Ub-Nrf2), (**D**) Kelch-like ECH-associated protein 1 (Keap-1), (**E**) superoxide dismutase-2 (SOD2) and (**F**) α-smooth muscle actin (α-SMA) are shown in mean ± SE of five mice for each group after calculating the intensity of the treatment relative to control ND and normalized by β-actin intensity. (**G**) 2-thiobarbituric acid reactive substances (TBARS) levels in liver are shown in mean ± SE of five mice for each group. Columns with different letters significantly differ (*p* < 0.05).

To evaluate the oxidative status in the liver, we measured the levels of some antioxidant factors and enzymes in the liver. The typical Western blots of three mice randomly selected from each group were shown in Figure 3A. The level of Nrf2, a key transcription factor that regulates the expression of antioxidant proteins, decreased to 0.7-fold in WD compared with ND, and recovered to 1.3-fold in WDBA (Figure 3B). Interestingly, the level of ubiquitinated Nrf2 (Ub-Nrf2) was increased to 3.6 in the NDBA group (Figure 3C). Additionally, Keap-1 (Figure 3D), a negative factor of Nrf2 by ubiquitination, showed the opposite result with Nrf2. The level of SOD2 (Figure 3E), a Nrf2 downstream target, also showed similar result with Nrf2. Next, we measured the level of liver TBARS, which is a secondary product of lipid peroxidation and has been widely adopted as a sensitive marker for lipid peroxidation in animal tissues [17]. As shown in Figure 3G, TBARS increased 6.4 times in the WD group compared with the ND group, and decreased significantly by supplementation with BA.

Finally, we assayed the level of α-SMA, an indicator of NAFLD-associated protein. The level was 1.3-fold higher in the WD group than that in the ND group, and decreased significantly to 1.0-fold by supplementation with BA (Figure 3F).

### 3.3. The Concentration of Caecal Organic Acids

To elucidate the effect of BA on gut environment, we measured caecal weight and short-chain fatty acids (SCFAs) concentrations. The caecal weight was significantly increased by supplementation with BA in both the ND and WD groups (Table 2). The concentration of lactic acid was significantly increased in both the ND and WD groups, and the concentration of butyric acid was significantly decreased in the NDBA group.

### 3.4. Modulation of Mouse Gut Microbiome

**Figure 4:**
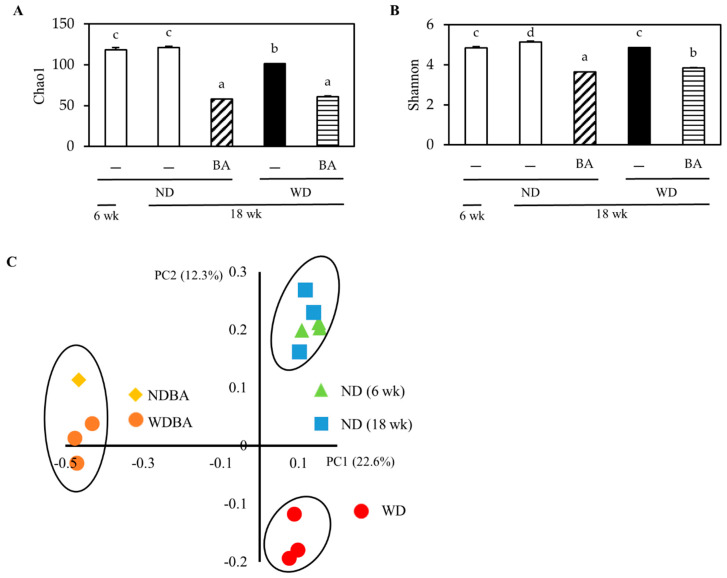
Effects of BA on total gut microbiome. The gut microbiome was characterized by 16S rRNA gene sequencing. The taxa richness of gut microbiome was assessed by α-diversity analyses using (**A**) Chao1 value and (**B**) Shannon index. These data represent the mean ± SE from each group. Columns with different letters differ significantly (*p* < 0.05). (**C**) The species compositions of gut microbiomes were assessed by β-diversity analyses using principal coordinate analysis (PCoA) of jaccard, which is showed in PC1 vs. PC2. Each dot represents the beginning (6 week) or ending point (18 week) of the experiment from each group.

To further understand the effect of BA on gut environment, the composition and relative abundance of gut microbiota were determined, using high throughput 16S rRNA gene sequencing. As shown in Figure 4A,B, Chao1 (species richness) and Shannon (species evenness) decreased in the WD group compared with the ND group. Supplementation with BA decreased them in both the ND and WD groups.

Furthermore, we used principal coordinate analysis (PCoA) plots (β-diversity: between-habitat diversity) based on Jaccard to investigate the similarities in gut microbial community structure among the different groups (Figure 4C). The percent of dataset variability explained by each principal coordinate is shown in the axis’s titles (PC1: 22.6%, PC2: 12.3%). The PCoA plot indicated that the structure of gut microbiota was divided 3 groups: (1) ND (6 weeks) and ND (18 weeks), (2) NDB and WDB (both at 18 weeks); (3) WD (18 weeks). These data suggested that the BA may regulate gut microbiome.

**Figure 5:**
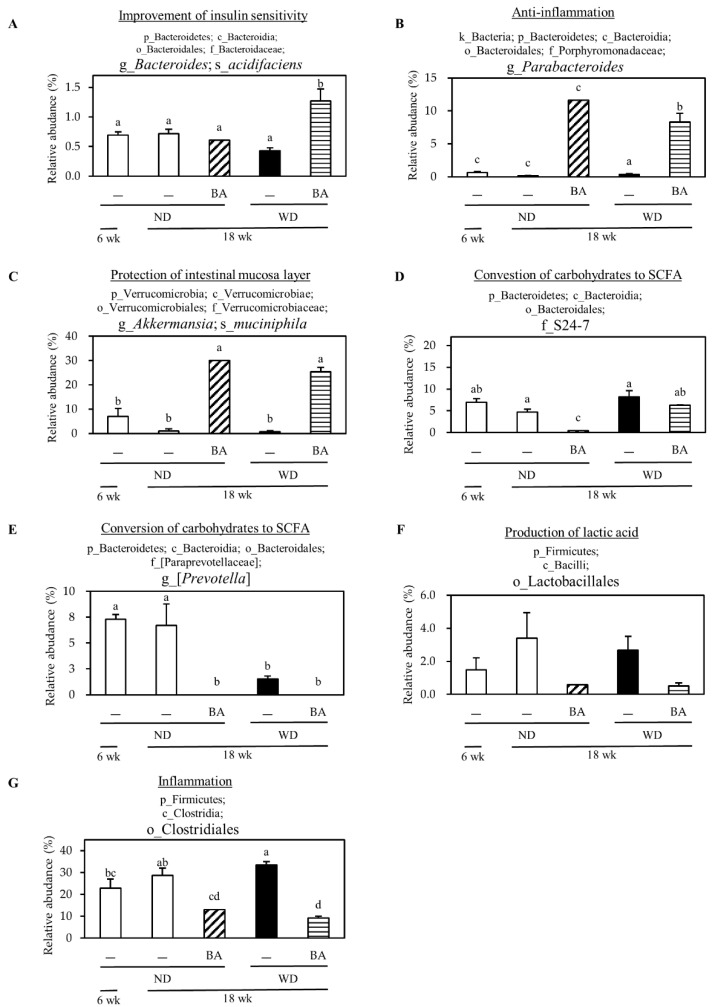
Modulation of BA on gut microbiome at the genus level. The data represent the relative abundance of each bacterial genus. p_, c_, o_, f_, g_, and s_ represent phylum, class, order, family, genus, and species respectively. (**A**) g_*Bacteroides*;s_*acidifaciens*, (**B**) g_*Parabacteroides*, (**C**) g_*Akkermansia*;s_*muciniphila*, (**D**) f_S24-7, (**E**) g_[*Prevotella*], (**F**) o_Lactobacillales, (**G**) o_Clostridiales. Columns with different letters significantly differ (*p* < 0.05).

Therefore, we further investigated the changes in individual microbial species at the phylum level (Table 3). The ratio of p_Firmicutes/p_Bacteroidetes (F/B) increased in WD, and significantly decreased by supplementation with BA. Moreover, BA significantly increased the abundance of p_Verrucomicrobia and decreased abundance of p_Deferribactere. Furthermore, BA significantly increased the abundance of g_*Bacteroides*;s_*acidifaciens* (Figure 5A), g_*Parabacteroides* (Figure 5B), g_*Akkermansia*;s_*muciniphila* (Figure 5C), and decreased the abundance of f_S24-7 (Figure 5D), g_*Prevotella* (Figure 5E), o_Lactobacillales (Figure 5F) as well as o_Clostridiales (Figure 5G).

### 3.5. A Correlation Between Gut Microbiota and NAFLD-Associated Factors

**Figure 6:**
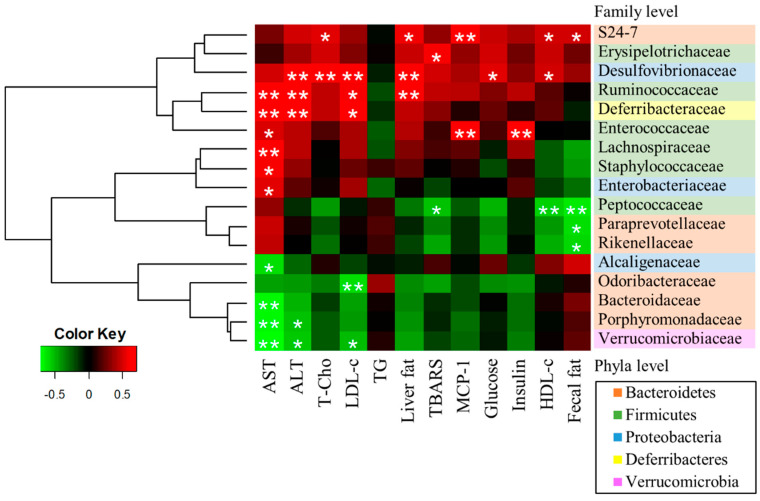
Heatmap of Spearman correlation between the alterations in gut microbial population and the changes in host parameters related to serum AST, ALT, T-Cho, LDL-c, TG, liver fat, TBARS, MCP-1, glucose, insulin, HDL-c, and fecal fat. * *p* < 0.05 and ** *p* < 0.01 were significantly negative (green) or positive (red) Spearman correlation.

To further explore the relationships between gut microbiome and NAFLD biomarkers, we generated a heatmap of Spearman correlations between gut microbial population and biomarkers (Figure 6). Both AST and ALT, the liver injury markers, were positively correlated with f_Ruminococcaceae, and f_Deferribacteraceae, and negatively correlated with f_Porphyromonadaceae and f_Verrucomicrobiaceae. T-Cho and LDL-c were positively correlated with f_ Desulfovibrionaceae. Liver fat was positively correlated with f_S24-7, f_Desulfovibrionaceae and f_Ruminococcaceae. TBARS was positively correlated with f_Erysipelotrichaceae, and negatively correlated with f_Peptococcaceae. MCP-1 was positively correlated with f_S24-7 and f_Enterococcaceae. Serum glucose level was positively correlated with f_Desulufovibrionaceae, and serum insulin level was positively correlated with f_Enterococcaceae. Fecal fat was positively correlated with f_S24-7, and negatively correlated with f_Peptococcaceae, f_Paraprevotellaceae; f_Rikenellaceae.

## 4. Discussion

In this study, bilberry anthocyanins showed preventive effects on WD-induced NAFLD by modulating serum biochemical markers, liver oxidative stress, caecal SCFA, and the gut microbiome structure and functions. In a previous study with the same bilberry anthocyanins extract (Mirtoselect), BA showed attenuation effects against the development of NASH by reducing hepatic lipid accumulation, inflammation and fibrosis [13]. Our data partially confirmed the effects and mechanisms of BA on NAFLD prevention in the previous report, and further suggested a novel mechanism that BA might prevent WD-induced NAFLD by modulating gut microbiome structure and functions.

BA has been reported to attenuate hepatic lipid accumulation although the serum markers of lipid metabolism were not investigated [13]. In the present study, we observed that BA attenuated significantly the serum levels of T-Cho (Figure 1D) and LDL-c (Figure 1F), and liver fat droplets (Figure 2) and fat amounts (Table 1). These data confirmed that bilberry anthocyanins extract could attenuate hepatic lipid accumulation as reported previously [13]. However, the previous study was designed to investigate the treatment effects of Mirtoselect on NASH [13] because all of the female E3L mice received WD for 4 weeks, and only the positive female E3L mice who matched the serum levels of cholesterol and triglycerides after 4 weeks WD intake were selected to further feed with Mirtoselect [13]. In the present study, we supplied BA in diet from the beginning of WD intake to estimate the preventive effects on NAFLD induction by WD. Thus, the symptoms of NASH in previous study was more critical than that of NAFLD in the present study, especially in inflammation and fibrosis. Additionally, our data also agreed with other reports in which blueberry polyphenol extract (anthocyanins compositions are similar as BA with lower contents [20]) inhibited serum LDL-c level, fecal TG and cholesterol production, but did not affect serum TG in high-fat diet (HFD)-fed C57BL/6 mice [21].

Oxidative stress is associated with the progression of NAFLD [22]. It has been reported that activation of Nrf2 inhibited NAFLD and NASH through upregulating heme oxygenase-1 (HO-1) /NAD(P)H:quinone oxidoreductase 1 (NQO1)/glutathione *S*-transferase (GST), then promoted hepatocellular carcinoma by competing with Keap1 or through FGFR4–GSK3β signal pathway [23]. In this study, we investigated the protein levels related to Nrf2/Keap1 antioxidant pathway. The results revealed that BA enhanced the levels of Nrf2 (a positive transcriptional factor) and SOD2 (a downstream antioxidant enzyme), and reduced the level of Keap1 (a negative transcriptional factor) and TBARS (a lipid peroxidation marker) in the liver. Therefore, the antioxidant action of BA may be involved in the preventive effects of NAFLD progression. On the other hand, it has been reported that the function of Nrf2 in lipid metabolism differs with age. In young Nrf2 knockout mice (12 weeks age) fed HFD (35% fat + 0.15% cholesterol) for 1 month, the expressions of hydroxymethylglutaryl-CoA reductase (a rate-limiting enzyme of cholesterol synthesis), acetyl-coenzyme A carboxylase (an enzyme for fatty acid synthesis), and sterol regulatory element-binding protein (a transcriptional factor of fatty acid synthesis) gene in liver were increased, compared with that in wild-type fed HFD [24]. However, in aged Nrf2 knockout mice (6 months age) fed HFD (35% fat + 0.15% cholesterol) for 3 months, their expressions were decreased [25]. In present study, all of biochemical indicators were measured at 18 weeks age, which belongs to young mouse. Therefore, an increase in Nrf2 level in WDBA might have a positive effect on the decrease in T-Cho (Figure 1D).

It has been reported that SCFAs have two major effects on host animals: one is to increase the calorie intake, another is to suppress the inflammatory reactions in the intestines [26]. In our study, lactic acid increased with BA administration (Table 2). Since lactic acid has a much lower pKa than acetic acid, propionic acid and butyric acid, thus, pH in the gut might be lower by BA administration. Lower pH is considered to be important factor in bacterial growth inhibition [27]. In fact, anthocyanins-rich food decreased butyric acid [28] and propionic acid in a rodent model [29]. Our data indicated that BA increased lactic acid, and decreased butyric acid. Therefore, BA administration may decrease harmful bacteria (e.g., o_Clostridiales) by selectively changing SCFAs composition.

The ratio of p_Firmicutes and p_Bacteroidetes (F/B) is an important marker representing the status of gut microbiome. The high ratio of F/B are often observed in obese and HFD-fed mice [30,31] with disrupted gut microbiome. In the present study, the ratio of F/B was significantly increased in the WD group, and reduced by BA administration (Table 3) These data demonstrated that BA attenuate WD-disrupted ratio of F/B in the gut microbiome. Moreover, BA increased the relative abundances of g_*Bacteroides*; s_*acidifaciens*, g_*Parabacteroides* (Figure 5B). It has reported that g_*Bacteroides*;s_*acidifaciens* may have potential for treating metabolic diseases such as diabetes and obesity [32], and g_*Parabacteroides*; s_*distasonis* attenuated toll-like receptor 4 signaling and Akt activation to block inflammation and colon tumor formation in the mice fed with HFD-azoxymethane diet [33]. Thus, BA may potentially have an anti-inflammatory effect by regulating these gut bacteria. Finally, BA significantly increased the relative abundances of g_*Akkermansia*; s_*muciniphila* (Figure 5C) g_*Akkermansia*, which is a Gram-negative, strict anaerobe and mucin-degrading bacterium [34]. Interestingly, treatment with g_*Akkermansia*;s_*muciniphila* could improve mucus layer thickness and metabolic disorders caused by HFD, including fat-mass gain, metabolic endotoxemia, adipose tissue inflammation, and insulin resistance [35]. These data suggested that BA may have protective effect on gut mucosa layer by enhancing the relative abundances of g_*Akkermansia*.

On the other hand, PCoA analysis of the gut microbiome showed that NDBA and WDBA groups were assigned in the same group. These data suggested that BA might also selectively regulate some gut bacteria in a diet-independent manner. A heatmap of Spearman correlation between the alterations in gut microbial population and the changes in host parameters revealed that both f_Porphyromonadaceae; g_*Parabateroides*, and f_Verrucomicrobiaceae; g_*Akkermansia* were negatively correlated with AST and ALT, serum markers of liver damage. It is known that f_Porphyromonadaceae; g_*Parabateroides* contributed to anti-inflammation [33] and f_Verrucomicrobiaceae; g_*Akkermansia* contributed to enhance mucus layer thickness [35]. Thus, it is possible that BA exert hepatic protective effects by increasing the relative abundance of these bacterial to protect the damage by intestinal toxins or inflammatory factors from enterohepatic circulation.

## 5. Conclusions

Bilberry anthocyanins ameliorated WD-induced NAFLD by attenuating gut microbiome dysbiosis and dyslipidemia. These data provide insights to fully understand the bioavailability and biological function of bilberry anthocyanins.

## Figures and Tables

**Table 1:** Organ weight and fat weight in liver and feces.

Index	ND		WD	
	—	BA	—	BA
Body weight (g)	35.3 ± 0.9 ^a^	33.3 ± 1.0 ^a^	40.7 ± 0.4 ^b^	35.8 ± 1.6 ^a^
Liver weight (g)	1.3 ± 0.1 ^a,b^	1.1 ± 0.0 ^a^	2.0 ± 0.1 ^c^	1.4 ± 0.1 ^b^
Epididymis fat weight (g)	1.7 ± 0.1 ^a,b^	1.3 ± 0.2 ^a^	2.2 ± 0.1 ^c^	1.9 ± 0.2 ^b,c^
Liver weight/body weight (%)	3.6 ± 0.2 ^a,b^	3.2 ± 0.1 ^a^	4.8 ± 0.2 ^c^	3.8 ± 0.1 ^b^
Epididymis fat weight/body weight (%)	4.8 ± 0.3 ^a,b^	3.9 ± 0.4 ^a^	5.5 ± 0.2 ^b^	5.3 ± 0.4 ^b^
Liver fat weight (%)	7.6 ± 1.2 ^a^	4.0 ± 0.4 ^a^	27.4 ± 2.9 ^b^	9.5 ± 1.8 ^a^
Total feces fat at 18 week (mg)	52.8 ± 7.2 ^a^	45.9 ± 2.4 ^a^	87.1 ± 4.9 ^b^	100.1 ± 4.4 ^b^

All of items were measured at 18 week. The numerical values with different letters (a, b and c) differ significantly (*p* < 0.05). ND: normal diet, WD: Western diet, BA: 2% bilberry anthocyanins.

**Table 2:** Caecum weight and short-chain fatty acids (SCFAs) composition in the caecum.

Index	ND		WD	
	—	BA	—	BA
Caecum (g/body weight, %)	0.64 ± 0.08 ^b^	1.04 ± 0.09 ^a^	0.69 ± 0.08 ^b^	0.96 ± 0.06 ^a^
Lactic acid (μmol/total cecum)	0.59 ± 0.08 ^a^	2.19 ± 0.49 ^b^	1.17 ± 0.20 ^a,b^	2.07 ± 0.49 ^b^
Acetic acid (μmol/total cecum)	3.92 ± 0.53	4.69 ± 0.71	2.84 ± 0.82	2.79 ± 0.16
Propionic acid (μmol/total cecum)	1.72 ± 0.49	1.95 ± 0.61	1.10 ± 0.16	1.17 ± 0.05
Butyric acid (μmol/total cecum)	0.66 ± 0.14 ^b^	0.21 ± 0.05 ^a^	0.77 ± 0.21 ^b^	0.55 ± 0.09 ^a,b^
Total SCFAs (μmol/total cecum)	6.88 ± 1.21	9.04 ± 1.66	5.89 ± 1.48	6.58 ± 0.46

The numerical values with different letters (a and b) significantly differ (*p* < 0.05). ND: normal diet, WD: Western diet, BA: 2% bilberry anthocyanins.

**Table 3:** Modulation of the gut microbiome at the phylum level.

Phylum Level of Gut Microbiome (%)	6 week	18 week			
	ND			WD	
	—	—	BA	—	BA
Firmicutes	37.0 ± 5.7 ^a^	42.6 ± 3.7 ^a^	17.6 ± 0.0 ^b^	48.3 ± 0.9 ^a^	12.5 ± 2.1 ^b^
Bacteroidetes	36.0 ± 3.6 ^b,c^	37.0 ± 4.3 ^b,c^	43.0 ± 0.0 ^a,b^	26.2 ± 2.7 ^c^	48.6 ± 2.3 ^a^
Verrucomicrobia	7.0 ± 3.3 ^b^	1.0 ± 0.9 ^b^	30.0 ± 0.0 ^a^	0.7 ± 0.4 ^b^	25.3 ± 1.9 ^a^
Actinobacteria	0.1 ± 0.0	0.9 ± 0.5	0.3 ± 0.0	0.9 ± 0.5	0.2 ± 0.1
Proteobacteria	16.4 ± 3.0	16.5 ± 4.2	9.1 ± 0.0	20.9 ± 1.2	13.4 ± 1.3
Deferribacteres	3.5 ± 0.7 ^a^	2.0 ± 0.9 ^a,b^	0.0 ± 0.0 ^a,b^	3.0 ± 1.1 ^a^	0.0 ± 0.0 ^b^
F/B	1.1 ± 0.3 ^b^	1.2 ± 0.2 ^b^	0.4 ± 0.0 ^b,c^	1.9 ± 0.2 ^a^	0.3 ± 0.1 ^c^

F/B means the ratio of Firmicutes/Bacteroidetes. The numerical values with different letters (a, b, and c) differ significantly (*p* < 0.05). ND: normal diet, WD: Western diet, BA: 2% bilberry anthocyanins.

## Appendix A

**Table A1:** Dietary compositions of each group.

Components (%)	ND		WD	
	—	BA	—	BA
Lard	3	3	30	30
Soybean oil	3	3	3	3
Corn Starch	45	43	16.5	14.5
Casein	20	20	20	20
Sucrose	20	20	20	20
Cellulose	4	4	4	4
Mineral Mix	3.5	3.5	3.5	3.5
Vitamin Mix	1	1	1	1
Cholesterol	0	0	1.5	1.5
Choline Bitartrate	0.2	0.2	0.2	0.2
Methionine	0.3	0.3	0.3	0.3
Bilberry anthocyanins (BA)		2		2
Total calories (kcal/100 g)	374	367	517	510

ND: normal diet, WD: Western diet, BA: 2% bilberry anthocyanins.
